# Liquid Organic Fertilizers for Sustainable Agriculture: Nutrient Uptake of Organic versus Mineral Fertilizers in Citrus Trees

**DOI:** 10.1371/journal.pone.0161619

**Published:** 2016-10-20

**Authors:** Belén Martínez-Alcántara, Mary-Rus Martínez-Cuenca, Almudena Bermejo, Francisco Legaz, Ana Quiñones

**Affiliations:** Department of Citriculture and Vegetal Production, Instituto Valenciano de Investigaciones Agrarias, Moncada, Valencia, Spain; MJP Rohilkhand University, INDIA

## Abstract

The main objective of this study was to compare the performance of two liquid organic fertilizers, an animal and a plant-based fertilizer, with mineral fertilization on citrus trees. The source of the fertilizer (mineral or organic) had significant effect in the nutritional status of the organic and conventionally managed mandarins. Nutrient uptake, vegetative growth, carbohydrate synthesis and soil characteristics were analyzed. Results showed that plants fertilized with animal based liquid fertilizers exhibited higher total biomass with a more profuse development of new developing organs (leaves and fibrous roots). Liquid organic fertilization resulted in an increased uptake of macro and micronutrients compared to mineral fertilized trees. Moreover, organic fertilization positively affected the carbohydrate content (fructose, glucose and sucrose) mainly in summer flush leaves. Liquid organic fertilization also resulted in an increase of soil organic matter content. Animal-based fertilizer, due to intrinsic composition, increased total tree biomass and carbohydrate leaves content, and led to lower soil nitrate concentration and higher P and Mg exchangeable in soil extract compared to vegetal-based fertilizer. Therefore, liquid organic fertilizers could be used as an alternative to traditional mineral fertilization in drip irrigated citrus trees.

## Introduction

Management techniques of agricultural production are nowadays focused on a greater commitment to environmental sustainability. On this way, organic agriculture, accepted by the EU and the FAO as an alternative system to conventional agriculture, appears to be an environmentally friendly growing system [1] since mineral fertilizers abuse and misuse are responsible for health problems and environmental pollution [2]. It has been claimed that organic agriculture is the fastest growing agriculture in the world. In the decade from 2001 to 2011, the total worldwide organic agricultural hectares (ha) grew by 135% [3], which equates to an 8.9% per annum compound growth over the decade. Ecological surface in Spain was 1845 10^3^ ha in 2011, representing an annual increase of 11.76% during the last decade, being positioned as the first country of the EU in number of ha in organic farming.

In organic systems, soil management involves the use of mowed or tilled cover crops, animal manures, composts and the application of organic fertilizers which increase soil-organic matter (SOM) whilst provide a steady release of nutrients to the crops as the organic matter breaks down. Exogenous organic matter applications are known to improve soil chemical and physical properties and biological functions, [4,5]. Ameliorated soil physical conditions enhance root growth facilitating nutrient uptake [6,7,8]. In this sense, organic manures have been demonstrated to improve the uptake efficiency of fertilizers [9]. Nutrient uptake efficiency may be defining as total element recovery in plant (mg) per fertilizer applied (mg). Moreover, the application of organic fertilizers has gained more importance not only because of its impact on soil quality but also because its role in carbon sequestration [10], mitigating atmospheric CO_2_ levels [11]. Organic fertilization has also been reported to have an influence on the phyto-nutritional quality of crops, enhancing the production of antioxidant metabolites in plants [12]. Yang et al. [13] obtained a remarkable increase in leaf yield, quality parameters, soluble sugar content, and thus in economic benefits, in *Stevia rebaudiana*, a perennial herb that it is an excellent source of sugars, under organic cultivation compared to traditional inorganic fertilization. In the medicinal plant *Labisia pumila*, organic fertilizer enhanced the production of total phenolics, flavonoids, ascorbic acid, saponin and gluthathione, when compared to inorganic fertilization [14]. However, liquid organic fertilizers to be used in fertigation with different origin are scarce and insufficiently tested.

Citrus (*Citrus* spp.) play an important role in organic farming system, being one of the most highly demanded products on the market for organic produce [15]. Nowadays, organic citrus makes about 2–7% of the global production [16] and 0.8% of the world citrus cultivated area [17], being thus a niche crop. In this context, the organic citrus sector has undergone a dynamic development in the last decade in the EU. The sector is concentrated around few Member States only: Italy, Greece, Spain and Cyprus. The biggest citrus areas are situated in Italy (more than 21900 ha) and Spain, where the sector amounted to around 6000 ha in 2011 and is under development.

In the past, citrus production was focused exclusively on maximizing the yield for commercial markets, and excessive fertilizer rates have been thus supplied to the crops with concomitant salt build up, phytotoxic effects on plant growth and ground water contamination [18,19,20]. In this context, efficient use of nitrogenous fertilizers has become a first-order concern in modern citrus production due to nitrate contamination of ground and surface waters [21,22]. Nowadays, fertilization studies are addressed to match and synchronize crop demand with nutrient supply. Interest in improving utilization of fertilizers, and specially N by citrus has been particularly widespread in Mediterranean areas, were citrus cultivation predominates [21,23,24], and most wells show nitrate concentrations clearly above the limit of the World Health Organization [25]. For nutrient uptake studies, the adoption of stable isotope techniques (^15^N) enables tracing the movement of fertilizer-N in the plant-water-soil system. Under organic fertilization ^15^N-labelled manure has allowed direct measurement and accurate estimation of N recovery not only in soil but also in crops [26]. But, due to the arduousness of the labelling procedure of organic residues and compost, very few assays on NUE of organic-derived fertilizers have been carried out, and all of them in horticultural crops [27,28,29,30].

The purpose of this study was to test the performance of two liquid organic fertilizers, a vegetal and an animal-based fertilizers, on citrus nutrient uptake, vegetative growth and soil characteristics, when compared to mineral fertilization under drip irrigation.

## Material and Methods

### Ethics statement

The experiments were conducted in the Department of Citriculture and Vegetal Production from Valencian Institute of Agrarian Research (Moncada, Spain). Dr. Ana Quiñones was response for experimental analysis in this manuscript and can be contacted in the future. The authors declare that this manuscript does not matter the any ethic issue and it does not involve endangered or protected species.

### Experimental conditions, plant material and treatments

The study was carried out in 2010/2011 at the experimental station of Valencian Institute of Agricultural Research in Moncada (39° 33' N; 24° 24' W; Valencia). Twenty homogeneous 4-year-old “Nules Clementine” mandarin (*Citrus reticulata* Blanco) with 18 cm of canopy diameter grafted on Carrizo citrange (*Citrus sinensis* x *Poncirus trifoliata*) rootstock were grown individually in 50 L pots containing a loam soil characterized by sand 45.1%, silt 38.1%, clay 16.8%; pH 8.4 with 0.37% total organic carbon concentration. At the beginning of the assay, the average canopy diameter at breast height (1 m above the soil surface) was 70 ± 8 cm and the diameter measured at 4 cm from the graft trunk area was 3.8 ± 0.3 cm for the rootstock and 2.8 ± 0.2 cm for graft. The containers were arranged outdoors on benches under polycarbonate shelter to exclude rain.

Two liquid organic fertilizers were tested, a vegetal (VO) and an animal-based (AO) fertilizer (Table 1). Forage maize (*Zea mays* L.) grown under ^15^N-labelled fertilizer supply was used as raw material for VO fertilizer production, and also as ^15^N-labelled sheep feed to obtain ^15^N-labelled manure. The labelled faeces fraction was used as raw material for the AO fertilizer. The VO fertilizer was obtained after an acidic and an enzyme-driven hydrolysis [31]. The AO fertilizer was obtained after acidic hydrolysis [31]. Liquid organic fertilizers (VO and AO) were compared to two mineral solutions containing 55 and 95% of N as ammonium sulphate and the remaining 45 and 5% as potassium nitrate, which were used as mineral controls for vegetal (VMC) and animal (AMC) fertilizer, respectively. The N fertilizer rate was 20 g N year^-1^·tree^-1^ according to tree canopy size [30], in order to fulfill plant N requirements.

**Table 1:** Total nitrogen and among fractions, ^15^N excess and macro and micronutrient concentration of plant (VO) and animal (AO) based organic fertilizer (mg·L^-1^) ^§^^†^.

	N total (mg·L^-1^)	N-NH^+^_4_ (mg·L^-1^)	N-NO^-^_3_ (mg·L^-1^)	NH_4_/NO_3_	N-organic (mg·L^-1^)	^15^N (atom % excess)
VO^††^	330.8±31.6	26.5±0.6^1^	21.6±0.03	1.23	282.6±6	2.62±0.07
AO^‡^	495.7±47.1	88.2±0.4	4.2±0.4	21.00	403.3±0.0	2.17±0.01
	C (mg·L^-1^)	C/N	P	K	Mg	Ca
VO	3504±43	10.6±1.43	47±1	923±14	60±2	386±9
AO	6046±801	12.2±2.3	365±26	921±65	257±19	545±28
	Fe	Zn	Mn	Cu	B	
VO	8.3±0.3	2.5±0.5	1.22±0.02	0.13±0.00	0.16±0.00	
AO	22.6±2.1	18.0±1.4	5.7±0.5	0.04±0.01	0.52±0.04	

^§^ Each value is a mean of three samples ± standard error.

^†^Volume applied: 60.5 and 40.3 L of vegetal and animal fertilizers, respectively.

^††^Ammonium and nitrate nitrogen accounted for 55 and 45% of released total inorganic nitrogen, respectively.

^‡^Ammonium and nitrate nitrogen accounted for 95 and 5% of released total inorganic nitrogen, respectively.

Other micro and macronutrients present in the vegetal and animal-based organic fertilizers were also supplied in similar amounts in their respective control. Nitrogen (N) and potassium (K) were supplied as potassium nitrate and ammonium sulphate. Phosphorus (P) fertilizer demand was applied as phosphoric acid and Magnesium (Mg) and Calcium (Ca) requirements as magnesium and calcium sulphate. The basic iron needs per tree were distributed throughout the growing cycle in a similar way to N in chelate form. Foliar spray treatments of zinc (Zn) and manganese (Mn) were applied as organic commercial fertilizer at 0.5% w/v (Zn: 6.6% w/w and Mn: 4.8% w/w) to correct deficiencies. In this way, organic fertilizers and their mineral controls differed in the nutrient form applied with the same concentration. Similar N and Ca amounts were added in all treatments (20 and 23 g.plant^-1^). However, higher K rate and lower P and Mg were applied with vegetal fertilizers (56 vs. 37, 3 vs. 15 and 4 vs. 10, g.plant^-1^ respectively). Plants nutritional requirements were supplied by treatments. Vegetal- and animal-based liquid organic fertilizers were ^15^N labeled (2.62 and 2.17%^15^N excess, respectively), while their respective controls were also labeled in the same extent. As a result, the experiment consisted of four treatments with five uniform trees per treatment, which were randomized across the experimental area. Mineral and organic fertilizers were supplied between March (spring growth resumption) and October. Plants were watered to field capacity every 2–3 days through 2 drip emitters per tree with deionized water, to avoid isotopic dilution of fertilizer-15N with water N. Mineral fertilizers were diluted into deionized water according to the following percentages [30], March (5%), April (10%), May (15%), June (22%), July (18%), August (15%), September (10%), October (5%). Similar quantities of organic fertilizers were manually added to each pot. The soil water potential was controlled daily using a ThetaProbe PR2 (Delta-T Devices, UK) and irrigation was scheduled when the matric potential at 30 cm depth attained -10 kPa [32,33].

With the aim of quantifying nutrient losses associated with abscised parts, tree litter (flowers, petals and fruitlets) was caught in nets from onset of flowering (1st April) until the end of fruit setting (4th July). Abscised organs were dried, weighed, milled and stored for subsequent nutrient and ^15^N analysis.

### Plant harvesting, sample preparation and vegetable analysis

At the end of the labelling period, during dormancy (December), trees were destructively harvested to determine nitrogen uptake efficiency (NUE) and nutrient plant uptake. Young (flowers/fruits, leaves and twigs of new shoots) and old organs (leaves and twigs of previous years, trunk and root system) were separated and sampled to quantify total dry biomass. All samples were washed in non ionic detergent solution followed by several rinses in deionized water, weighed, frozen into liquid nitrogen, freeze-dried and dry-weighed.

Vegetal samples were ground with a water-refrigerated mill, then sieved through a 0.3 mm mesh sieve and stored at -20°C for further analysis.

Macronutrients (P, K, Ca, Mg) and micronutrients (Fe, Zn, Mn) were measured in simultaneous inductively coupled plasma atomic emission spectrometry (ICAP-AES 6000, Thermo Scientific, Cambridge, United Kingdom) [34], after nitric-perchloric digestion. Dried plant material (0.5 g) was pre-digested overnight with 10 mL HNO_3_ on a digestion block at 120°C. The samples were cooled down to room temperature and 2.0 mL of a 70% ultra-trace-metal-grade HClO_4_ was added and re-digested at 220°C until white fumes were produced. Digest product was diluted to 25 mL with ultrapure water [35] and nutrient concentrations were subsequently measured.

Determinations of total N and C concentration and ^15^N abundance were performed with an Elemental Analyzer (NC 2500 Thermo Finnigan) coupled to an Isotope Ratio Mass Spectrometer (Delta Plus, Thermo Finnigan). Results were expressed as percentage (macronutrient) or parts per million (micronutrients) of dry weight (DW).

### Carbohydrates determination: soluble sugars in leaves

Leaf samples were washed, lyophilized, ground and stored at 4°C. Soluble sugars were extracted (100 mg DW) with 5 mL of ethanol 80% (v/v) at 60°C for 15–30 min, and then mixtures were centrifuged at 10000 rpm, for 30 min at 4°C. For recovery purposes, known amounts of fucose (Sigma Quimica, Madrid, Spain), a sugar absent in the extracts, were added to extracts as an internal standard. The supernatant was removed and the pellet was extracted twice (the extraction was repeated three times). The combined supernatants were collected, evaporated in vacuo at 45°C [36]. Residues were redissolved in 1 mL of water, and then filtered through 0.45 μm nylon filter and analyzed by HPLC by refraction index, using a column Tracer Carbohydr 250 mm x 4.5 mm, 5 μm (Teknokroma, Barcelona, Spain) and a mobile phase composed by acetonitrile:water (75:25) at a flow rate of 1 mL min^-1^. An HPLC system equipped with a Waters 515 HPLC pump, a Waters 2414 refractive index detector and a 20 μL loop Rheodyne injector was used for sugar analysis. Empower 2 software (Waters, Spain) was used for data processing. Fructose, glucose and sucrose sugars were identified by comparing their retention time with a standard and quantified using an external calibration curve.

### Soil sampling, sample preparation and analysis

On July, when half of the N was supplied, soil samples were extracted with a stainless steel cylindrical auger of 4 cm in diameter. At the end of the experiment, on December, soil contained in the pot of each harvested plant was thoroughly mixed after completely removing the fine roots, and then weighed before sampling. Each sample consisted of three subsamples, which were air-dried at room temperature, dry weighed, crushed through a 2 mm screen and stored for analysis.

The mineral nitrogen of the soil (NO_3_-N and NH_4_-N) was measured with flow-injection analysis (FIAstar 5000, Foss Tecator, Höganäs, Sweden) in KCl extracts (2 M), according to Raigon et al. [37]. In order to determine ^15^N/^14^N isotopic composition of both mineral fractions, soil extracts were steam-distilled (2200 Kjeltec, Auto Distillation Unit, Foss Tecator, Höganäs, Sweden); ^15^NH_4_-N and ^15^NO_3_-N were recovered in boric acid [38]. Aliquots were acidified with 0.32 N H_2_SO_4_ and reduced to dryness in an oven (P Selecta, Barcelona, Spain) at 65°C before analysis using the mass spectrometer mentioned above. After KCl extraction, the residual soil samples were washed with 50 mL distilled water to remove the remaining extractant solution, air-dried, milled, and analyzed for the organic N concentration and ^15^N/^14^N isotopic composition [39] using the Elemental Analyzer coupled to an Isotope Ratio Mass Spectrometer aforementioned.

### Calculations

Based on data of dry weight (DW, g) and total nutrient concentration ([Nutrient], %, w/w) for each plant compartment, nutrient content was calculated:

$$Nutrient\left(g\right)\;=\;\frac{\left[Nitrogen\right]\;\times \;DW}{100}$$

The ^15^N content per plant compartment was calculated as follows:

N15plant compartment(mg) = [Nitrogen] × DW × atom N15excess10

Where *atom* ^15^*N*_*excess*_ was calculated by subtracting the natural abundance of ^15^N from the atom % ^15^N in each sample. The natural abundance of ^15^N was considered to be the abundance of atmospheric N_2_, 0.3663 atom %, according to the International Atomic Energy Agency [40].

The fraction N which is derived from the fertilizer (Ndff) was calculated according to Hardarson [41]:

Ndff(%) = 100 × atom %N15excess plant compartmentatom %N15excess fertilizer

Total plant recovery of applied ^15^N-fertilizer represents the proportion of applied ^15^N that is taken up by the tree and embodies its fertilizer-nitrogen uptake efficiency (NUE). NUE was calculated by the formula:

Ndff(%) = 100 × atom %N15excess plant compartmentatom %N15excess fertilizer

NUE(%) = 100 × N15whole plant(mg)N15fertilizer(mg)

Where:

N15whole plant(mg) = ∑N15(mg)plant compartments

The amount of ^15^N recovered in each soil N fraction (Organic-^15^N, NO_3_-^15^N, NH_4_-^15^N) was determined as follows:

N15soil fraction(mg) = Nsoil fraction(mg Nkg soil) × soil DW (kg) × N15excess100 

Then, nitrogen retained in soil profile was calculated by the formula:

Ndff soil = 100 × N15soil fraction(mg)N15fertilizer(mg)

### Statistical Analysis

Data were subjected to ANOVA to test for significant differences between treatments. Before carrying out any statistical analysis, the normality of all the data was studied using the Kolmogorov-Smirnov test. In case the hypothesis of normality was discarded at the 95% confidence level, the data were transformed according to the logarithmic function. Otherwise, the data analyses were carried out with the variables measured in their natural scales. The variance of the transformed or non-transformed data was partitioned through a variance analysis (ANOVA, Statgraphics Centurion for Windows, Statistical Graphics Corp.) into one source of variability. The experiment consisted of two factors i) the source of the nutrients, organic versus mineral fertilizers, ii) the origin of organic fertilizer (vegetal- or animal-based). The significance of the comparisons made among treatments was analyzed using Fisher’s least significance difference (LSD) test at P < 0.05.

## Results and Discussion

### Plant Biomass

**Fig 1:**
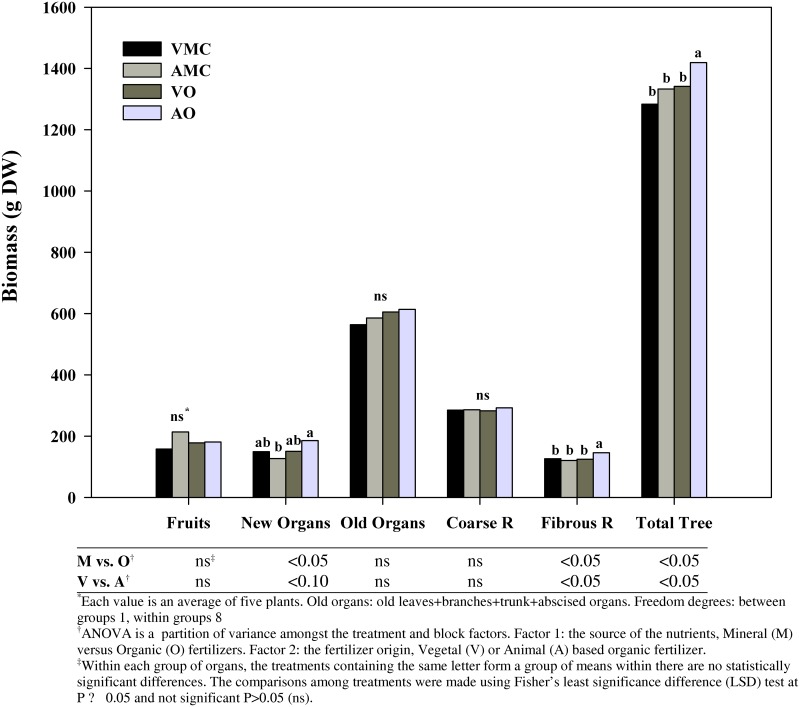
Distribution of dry biomass (g DW plant^-1^) among the main organs of Nules clementine trees harvested in December (at dormancy) receiving vegetal (VO) or animal-based (AO) organic fertilizer and their respective mineral controls (VMC and AMC, vegetal and animal mineral controls, respectively)*.

Different sources of nutrient (organic or mineral) had significant effect on total tree biomass, in spite of an equal N dosage per tree was applied in all treatments (Fig 1). Plants fertilized with organic fertilizers showed higher total biomass than mineral fertilized trees, as a result of a more profuse development of new organs and fibrous roots. Plant growth stimulation under organic management has been previously reported.

In citrus, enhancements of the physical growth characters (trunk and shoot diameter, shoot length, tree canopy and leaf area) have been recorded under organic fertilization based either on poultry manure [42] and compost plus humic acid [4] when compared to mineral NPK fertilizers. Also in peach trees, plant biomass was enhanced by supplying different organic fertilizers [6]. Biomass stimulation is the consequence of the hormone like effect that humic acids present in organic fertilizers play on the whole plant and especially on root growth [6,43]. In this sense, Hassan et al. [44] with mineral nitrogen plus humic acid as soil application observed higher leaves dry weight per plant than the other treatments in olive.

According to the origin, animal-based organic fertilizer led to a greater total biomass than vegetal-derived, due to the enhancement of new organs and fibrous roots development. However, Baldi et al. [45] found organic fertilization improved plant biomass, without differences between cow manure and compost obtained from municipal solid waste mixed with pruning material. Nevertheless, it is worth mentioning that in this experiment animal manure was pre-digested in order to obtain a liquid fertilizer, which would have increased nutrient availability compared to a direct application of cow manure.

### Nitrogen uptake efficiency

**Fig 2:**
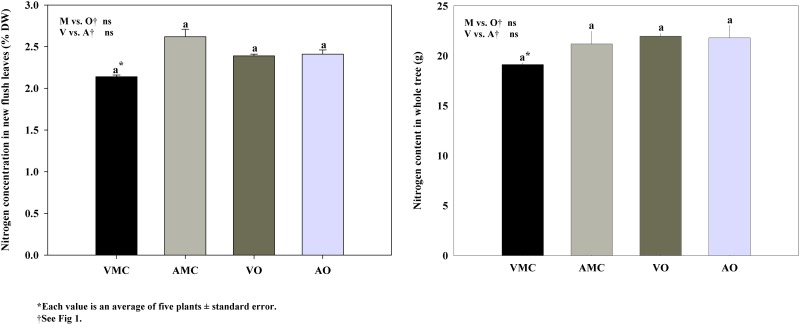
Nitrogen concentration (a) in new flush leaves (NFL) and total content in whole tree (b) of Nules clementine trees harvested in December (at dormancy) receiving vegetal (VO) or animal-derived (AO) organic fertilizer and their respective mineral controls (VMC and AMC, vegetal and animal mineral controls, respectively)*.

Foliar nitrogen values were in the optimal range according to the standards for citrus nutritional status diagnosis established by Quiñones et al. [30] without significant differences between treatments (Fig 2a). No significant differences were also observed in nitrogen content in whole tree (Fig 2b). However, other authors found a effect of concentration and source of N added (NO_3_^−^ or NH_4_^+^) on nutrient concentrations in leaves from citrus trees [46,47]. Nitrogen concentrations in leaves were highest when plants were provided with either NO_3_^−^ or NH_4_^+^ as a unique source of N. However, lowest N concentration in leaves was found with a 75:25 NO_3_^−^:NH_4_^+^ratio.

**Fig 3:**
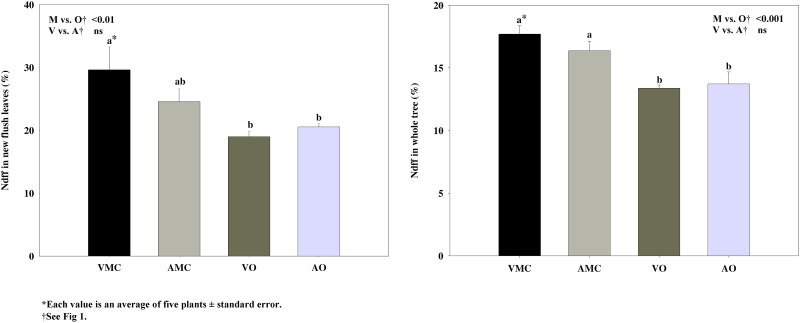
Nitrogen derived from fertilizer (Ndff, %) in (a) new flush leaves (NFL) and (b) whole tree of Nules clementine trees harvested in December (at dormancy) receiving vegetal (VO) or animal-derived (AO) liquid organic fertilizer and their respective mineral controls (VMC and AMC, vegetal and animal mineral controls, respectively)*.

Nitrogen derived from fertilizer (Ndff), that is the relative contribution of fertilizer N to the total content of this element in plant organs, was lower in new flush leaves (Fig 3a) and on the average of the whole tree (Fig 3b) under organic fertilization when compared to mineral fertilization at the end of the cycle. This lower contribution of fertilizer-N to the total content of this element results from the diminished availability of N from fertilizer [48], since more than 80% of the total N supplied in the vegetal and animal-based liquid fertilizers was in organic form. The fact of the lower contribution of fertilizer-N in organic than in mineral supplied trees, together with the lack of differences in total N concentration among treatments, points to an enhanced remobilization of reserve N in organically fed trees. Similar result was found in nitrogen uptake efficiency (NUE) in whole tree (Table 2) with slightly greater values in plants in which mineral fertilizers were supplied. Bosshard et al. [49] found a similar pattern, 37, 10 and 47% of ^15^N applied as urine, faeces and mineral fertilizer were recovered, respectively, in wheat.

**Table 2:** Nitrogen uptake efficiency (NUE) in whole tree and nitrogen recovered in different soil fractions of Nules clementine trees harvested in December (at dormancy) receiving vegetal (VO) or animal-derived (AO) liquid organic fertilizer and their respective mineral controls (VMC and AMC, vegetal and animal mineral controls, respectively) ^§^.

	VMC	AMC	VO	AO	M vs. O^†^	V vs. A^‡^
NUE	33.87±1.5a^††^	34.47±0.50a	29.90±0.90b	29.59±0.21b	**	ns
Soil NH_4_-N	0.33±0.04bc	0.26±0.04c	0.58±0.07a	0.49±0.08ab	**	ns
Soil NO_3_-N	12.32±1.19a	8.37±1.16b	1.53±0.14c	1.17±0.17c	***	**
Soil Organic-N	14.08±0.69b	13.87±0.80b	32.05±2.41a	30.90±1.39a	***	ns

^§^Each value is an average of five soil samples plants ± standard error.

^†^ANOVA. Partition of variance amongst the treatment and block factors according to Fisher’s least significance difference (LSD) test at P ≤ 0.05 (*), P ≤ 0.01 (**), P ≤ 0.001 (***) and not significant P>0.05 (ns). Factor 1: the source of the nutrients, Mineral (M) versus Organic (O) fertilizers

^‡^Factor 2: the organic fertilizer origin with two levels, Vegetal (V) or Animal (A) based organic fertilizer.

^††^Within each row, different letters denote differences among means according to Fisher’s least significance difference (LSD) test at P ≤ 0.05 and not significant P>0.05 (ns).

Neither Ndff (Fig 3a and 3b) in new flush leaves and in the whole tree nor NUE (Table 2) were affected by the origin (vegetal or animal based) of the liquid organic fertilizers tested.

### Macro and micronutrient content and carbon fixation in plant organs

**Fig 4:**
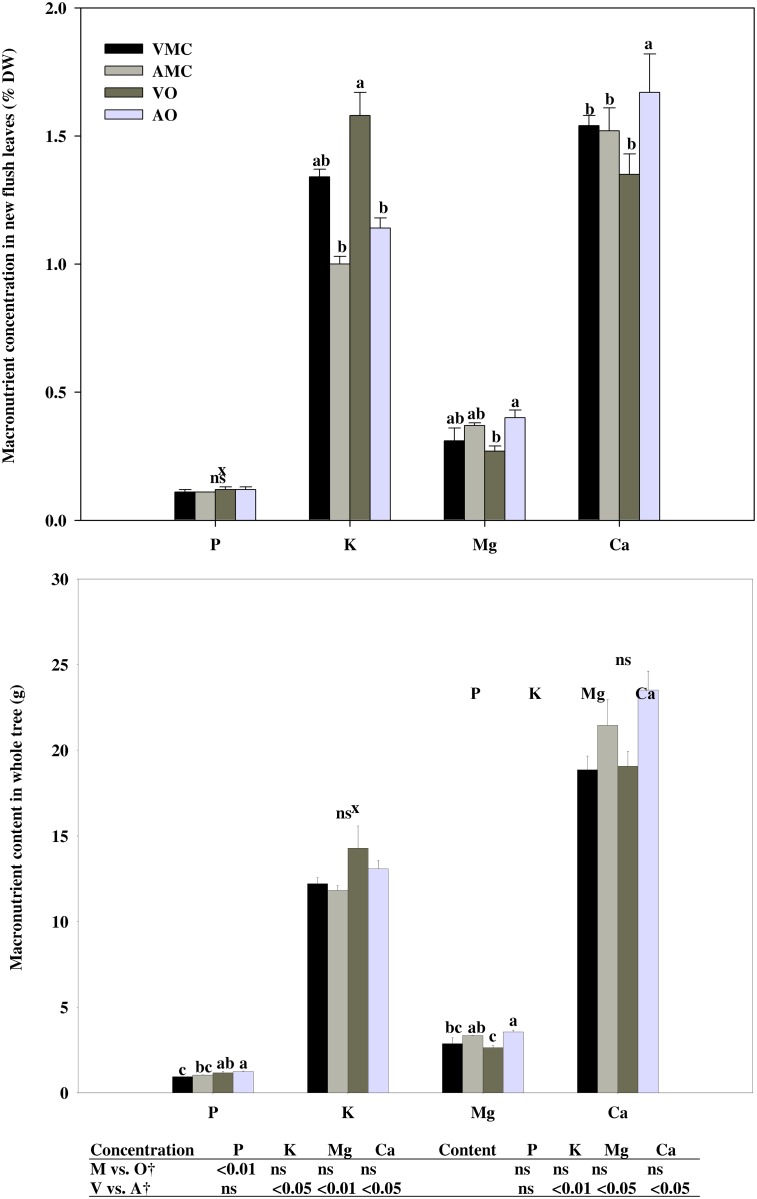
Macronutrient concentration (% DW) in new flush leaves (a) and total content (g) in whole tree (b) of Nules clementine trees harvested in December (at dormancy) receiving vegetal (VO) or animal-derived (AO) organic fertilizer and their respective mineral controls (VMC and AMC, vegetal and animal mineral controls, respectively)*. *, ^†^See Fig 1.

**Fig 5:**
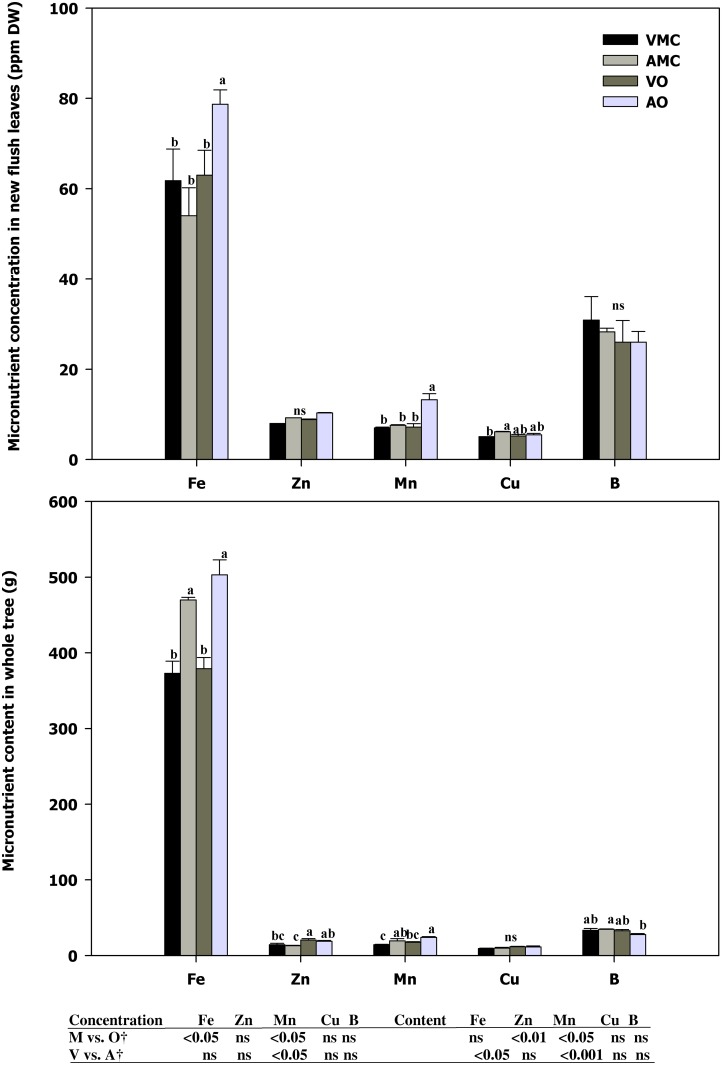
Micronutrient concentration (% DW) in new flush leaves (a) and total content (g) in whole tree (b) of Nules clementine trees harvested in December (at dormancy) receiving vegetal (VO) or animal-derived (AO) organic fertilizer and their respective mineral controls (VMC and AMC, vegetal and animal mineral controls, respectively)*. *, ^†^See Fig 1.

According to the analysis of spring leaves, the source of the fertilizer (mineral or organic) had a significant effect on the nutritional status of the organic and conventionally managed citrus trees. Phosphorous (P) content was higher in organic management, mainly due to both slightly higher concentration and greater biomass developed in plants fertilized with organic compost (Fig 4a and 4b). In micronutrients (Fig 5a and 5b), a significantly lower iron (Fe), manganese (Mn) concentration and zinc (Zn) and Mn content were found in plants fertilized with mineral fertilizers.

Serna et al [47] found different behaviour with increasing proportions of NH_4_^+^ in the N supply, where leaf nutrients such as P and Fe increased, whereas Ca, K, Mn and Zn decreased. No consistent results with this respect have been found in the present study probably due to the fact that about 80% of total N supplied was in organic form.

These results are similar to that found by Barakat et al. [4] on Newhall and Canali et al. [15] on Tarocco and Navelina oranges on a 4-year average investigation, in citrus. These authors showed an improvement on plant nutritional status with organic fertilization compared to the chemical fertilizers through increasing their contents of phosphorus and potassium. Helail et al. [50] also showed an enhancement in leaf mineral content by application of organic manure of Washington Navel orange. Baldi et al. [51] observed no differences in Ca, Mg and Fe accumulation in relation to non-composted trees. However, Canali et al. [52] on Valencia late grafted on sour orange and Gasparatos et al. [53] on apple trees showed no significant difference between treatments for leaf N, K and P content whereas significant differences for Ca, Mg and micronutrients were observed. These different results may be due to the broad variety of organic fertilizers and differences in nutrient compositions depending on the origin of the fertilizer, moreover, differences in physical properties of the animal or vegetal subproducts result in a great variability in solubility and thus in elements availability for plant uptake [54]. As shown above, in comparison with mineral fertilizers, they provide lower levels of N, P and K; however, their addition can provide minerals to the soil (calcium, sulfur, iron, boron, and zinc), which will be continuously available to the growing plant. Unlike, all these elements are not supplied when unique mineral fertilizers are added [10].

Concerning to the origin of the organic fertilizers (vegetal vs. animal-based), significant differences were observed on K and Mg concentration according to the different mineral composition of both organic fertilizers. Vegetal-based fertilizer supplied higher amounts of K whereas in animal-based fertilizer Mg content was reinforced. In the case of Ca, the amount supplied was similar in both treatments. However, calcium uptake was higher (concentration and content) in plants receiving the animal-based fertilizer. This finding is attributable to a greater availability of calcium in faeces than in vegetal-based fertilizer. Canali et al. [51] found no differences in macronutrient concentration in Valencia late fertilized with citrus by-product compost (as vegetable fertilizer) and poultry manure (as animal manure) and Baldi et al. [45] also found similar N and P concentration in plant addressed with compost and cow manure, but with higher K leaf concentration in plant fertilized with compost. This response was probably due to the great K amount of K added with compost fertilization that inhibited Ca and Mg uptake. Moreover, K concentration was lower in compost fertilizer than in cow manure but with a master release of K. In this assay, as said above, manure was previously digested which increased their availability.

However, all values for macro and micronutrients were in the optimal range according to the international standard citrus nutritional status [30].

**Fig 6:**
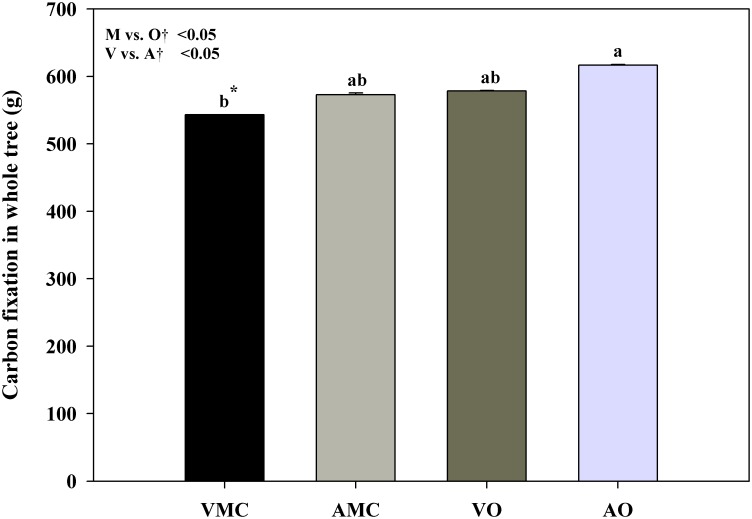
Carbon fixation in whole tree of Nules clementine trees receiving vegetal (VO) or animal-based (AO) organic fertilizer and their respective mineral controls harvested in December (at dormancy). Each value is an average of five plants ± standard error. ^†, ‡^See Fig 1.

With respect to carbon fixation, total carbon in trees supplied with organic fertilizers was higher than in conventional fertilization, and greater when liquid animal manure was applied (Fig 6), these results logically paralleled those of tree biomass. Similar results were obtained by Baldi et al. [51] in nectarine.

### Soluble foliar carbohydrates content

In order to determine the carbohydrates content according to the fertilizer applied, soluble sugars were analyzed in leaves from different flushes (spring and summer flush and old leaves). Under organic fertilization, soluble carbohydrates contents were significant greater in summer flush leaves and slightly higher in the other sproutings than conventionally fertilizer plants (Table 3).

**Table 3:** Soluble carbohydrate contents (g 100 g^-1^ DW) among the main flushes of Nules clementine trees harvested in December (at dormancy) receiving vegetal (VO) or animal-derived (AO) liquid organic fertilizer and their respective mineral controls (VMC and AMC, vegetal and animal mineral controls, respectively) ^§^.

	Carbohydrate	VMC	AMC	VO	AO	M vs. O^†^	V vs. A^‡^
Spring flush leaves	Fructose	0.61±0.13a^††^	0.82±0.18b	0.64±0.21bc	1.10±0.27c	*	**
	Glucose	0.64±0.18b	0.44±0.104a	0.45±0.13a	0.70±0.13b	ns	ns
	Sucrose	1.77±0.47b	1.97±0.59b	1.84±0.21b	2.01± 0.58c	ns	ns
Summer flush leaves	Fructose	0.42±0.08a	0.58±0.18a	0.49±0.11ab	0.72± 0.14b	**	*
	Glucose	0.37±0.06a	0.4 ± 0.08a	0.44± 0.10a	0.84± 0.33a	**	**
	Sucrose	0.82±0.30a	1.11±0.43a	1.13±0.26a	1.62± 0.49b	*	*
Old leaves	Fructose	0.47±0.06a	0.40±0.03a	0.42±0.06a	0.55±0.08a	ns	ns
	Glucose	0.42±0.06a	0.39± 0.06a	0.36± 0.04a	0.45±0.08a	ns	ns
	Sucrose	0.60±0.05a	0.81± 0.05a	0.75± 0.03a	0.77± 0.20a	ns	ns

^§^Each value is an average of five plants ± standard error.

^†, ‡,††^See Table 2.

Other authors have reported higher levels of primary metabolites and phenolic contents in horticultural cultivars, strawberries [55], marionberries [56], apples [57] and sweet pepper [58], among others, grown under organic conditions compared with inorganic fertilization. Generally, these results suggest that the usage of organic fertilizers can enhance the production of secondary and primary metabolites, and positively affect carbohydrate content. Zekri and Obreza [59] founded a positive influence of Magnesium fertilization on the synthesis of carbohydrates in leaves. Higher soluble carbohydrate content in leaves may be due to a greater Mg rate applied with animal derived liquid fertilizer (O). Moreover, source of the organic fertilizer also affected on the sugars content, mainly in summer flush leaves, with higher values when animal-based fertilizer was supplied. Summer leaves showed the most remarkable differences since according to timing of fertilizer application, these flush sprouts when approximately 50% of the total fertilizer rate has been supplied. No differences were found in old leaves that developed the prior year.

### Soil characteristics and N recovered

The analysis of soil characteristics at the end of the growing cycle indicated higher values of soil organic matter (SOM) and N, P, Ca and Mg exchangeable in those soils corresponding to organic fertilized plants, compared to mineral fertilized (Table 4). No significant differences in pH, and electrical conductivity (EC), K, and Na exchangeable were found among mineral and organic fertilized soils, with similar values to other agricultural fields. Numerous authors have found K deficiencies under organic fertilization management [51,60,61]. But the lower values of these variables under organic management system seem to be due to an insufficient input of organic sources [51]. In this assay, K rate was similar in both managements (organic or mineral) being available for plant uptake.

**Table 4:** Effect of vegetal (VO) or animal-derived (AO) liquid organic fertilizers and their respective mineral controls (VMC and AMC, vegetal and animal mineral controls, respectively) on soil characteristics at the end of the growing cycle^§^.

Analysis	Initial	VMC	AMC	VO	AO	M vs. O^†^	V vs. A^†^
pH	8.4±0.01	8.2±0.0b	8.1±0.0b	8.5±0.0a	7.9±0.0	ns	ns
EC (dS·m^-1^) ^‡^	0.25±0.03	0.66±0.03b	0.81±0.02b	0.83±0.09b	0.82±0.01a	ns	ns
C (%)	0.37±0.02	0.36±0.02b	0.36±0.00b	0.50±0.02a	0.48±0.02a	***	ns
N (% dw) ^††^	0.04±0.00	0.04±0.00b	0.04±0.00b	0.05±0.01a	0.05±0.00a	**	ns
C:N	9.25±0.12	9.00±0.25b	9.00±0.14b	10.00±0.08a	9.6±0.18a	**	ns
P (mg·kg^-1^ dw) ^‡‡^	13.5±0.7	27.5±1.5b	30.5±0.5b	31.7±0.9b	64.3±8.5a	*	*
K exchange^•^	0.27±0.02	0.95±0.05a	0.71±0.01b	1.03±0.04a	0.52±0.03c	ns	***
Mg exchange^•^	1.27±0.03	1.31±0.07c	1.77±0.01ab	1.71±0.03bc	2.18±0.18a	*	**
Ca exchange^•^	4.89±0.69	4.32±0.78a	5.56±0.10a	4.76±0.27a	6.04±0.35a	*	*
Na exchange^•^	0.23±0.01	0.34±0.01a	0.33±0.01a	0.46±0.11a	0.21±0.06a	ns	ns

^§^Each value is an average of five soil samples plants ± standard error.

^†^See Table 2

^‡^Electric conductivity in ext. 1:5 H_2_O at 25°C.

^††^Total Nitrogen Kjeldahl.

^‡‡^Phosphorous Olsen.

^•^Exchangeable cation in meq·100 g^-1^.

In general, most agricultural benefits from compost application to soil are derived from improved physical properties related to increase SOM rather than its value as a fertilizer [4,62] and is directly related to soil quality [63,64]. Initial results of the introduction of organic farming on soil quality of organically managed citrus orchards in the Mediterranean region were reported by Intrigliolo et al. [65]. They reported that organic management induced only slight differences in the main physical and chemical characteristics of conventionally managed soil. However, Canali et al. [52], in their study based on the comparison between organic and conventional citrus orchards, found significant differences in SOM content and soil aggregates stability, and no significant differences were found for clay, sand, pH and EC [13].

**Fig 7:**
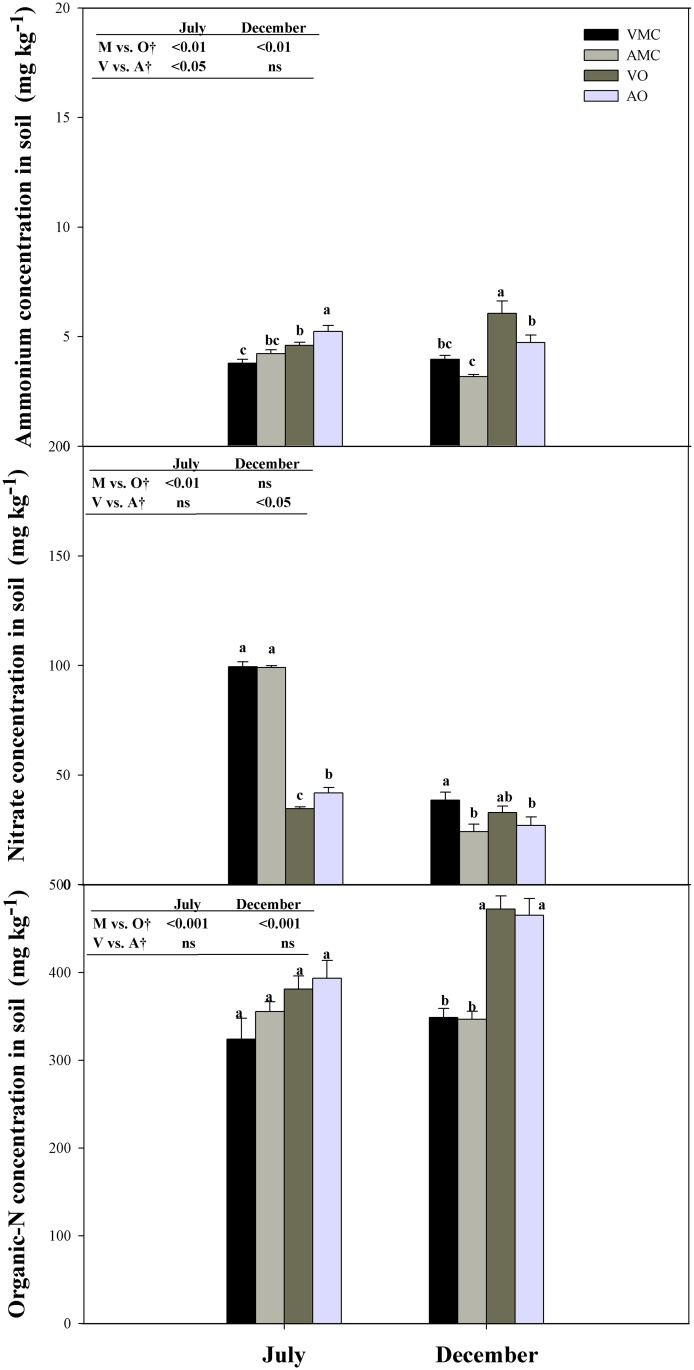
Nitrogen concentration in different N fractions in soils receiving vegetal (VO) or animal-based (AO) organic fertilizer and their respective mineral controls (VMC and AMC, vegetal and animal mineral controls, respectively)*. *, ^†, ‡^See Fig 1.

Nitrogen fractions in soil samplings on July and December revealed differences due to the source of the fertilizer applied (Fig 7). Organic fertilization led to greater concentrations on N in the ammonium and organic fractions in both sampling events. However, nitrate-N concentration in soils fertilized with organic liquid fertilizers was almost one third of that in mineral fertilized soils on July. Nevertheless, at the end of the cycle, once the nitrate supplied has been preferentially uptaken, differences in nitrate concentration were almost negligible because of the slow nitrification of the ammonium-N forms.

The origin of liquid organic fertilizers also affected nitrogen partitioning among soil N pools. Animal-based fertilizer resulted in an increased concentration of N present in the ammonium fraction on July sampling event as a consequence of a reinforced supplied of ammonium-N form if compared to vegetal-based organic fertilizer. At the end of the cycle nitrate-N concentration was higher in plants supplied with vegetal-based fertilizer due to the nitrification of ammonium-N supplied.

Similarly, the percentage of fertilizer-N recovered in different soil-N fractions at the end of the assay (Table 2) showed that the amount retained as nitrate-N was significantly higher under mineral fertilization when compared to organic fertilization (10.35% and 19.15%, respectively). The reverse pattern was found in organic-N, with organic fertilized soils exhibiting the greater values (1.35% in mineral and 31.07% in organic fertilization). No differences were found on fertilizer-N compartmentation in soil N fractions attending to the origin (animal or vegetal) of the organic liquid fertilizer. Soil residual nitrate-N was higher in the plants supplied with the VMC than in those receiving AMC where N was predominantly (95%) in ammonium form. Nevertheless, no differences in NUE were found probably due to the fact that in VMC N was equally supplied under ammonium (55%) and nitrate (45%) forms. This lower residual NO_3_-N accumulation under organic management is advantageous in comparison with conventional fertilization from an environmental point of view. Numerous authors linked main source of groundwater pollution with NO_3_ leached from intensive agricultural areas [66,67]. So, the application of organic fertilizers can enhance nutrient uptake, mainly N, by reducing mineral leaching. Moreover, some nutrients in the water-soluble form required by plants are readily leached from mineral soil particles, whereas they are effectively held on the surface of humified organic matter [10]. The pre-digestion of organic residues in order to obtain water soluble fertilizers leads to an equivalent availability of nutrient elements to that in traditional mineral fertilizers.

It is worth mentioning that only a small fraction of the labeled fertilizer N was recovered as ammonium in all treatments, being higher in organic fertilized plants. This can be due to the process of fertilizer nitrate immobilization and later mineralization of labelled soil organic matter [24]. Davidson et al. [68] found a rapid turnover of a small NO_3_-pool in intact soil cores due to a rapid phase of immobilization immediately following the addition of ^15^N tracers to soils. The labelled ammonium-N found at the end of the trial accounted for less than 1% of applied-^15^N for all treatments.

## Conclusion

Liquid organic fertilizer obtained from maize residues (vegetal-based) and faeces sheep manure (animal-based) promoted biomass production and nutrient concentration in citrus plants. Organic fertilizer also resulted in an increase of soil organic matter. Moreover, organic fertilization positively affected the carbohydrate content. Plant fed with animal-based fertilizer, due to intrinsic composition, displayed a better biomass development and mineral nutrition.

The presented data support the idea that liquid organic fertilizers can be successfully used as a substitute of mineral fertilizers in citrus trees nutrient management under drip irrigation, since they enhance soil chemical fertility, prevent excessive nitrate-N concentration, promote plant growth and C fixation in the plant. Moreover, these fertilizers would allow not only to reduce the use of chemicals, but also to re-use crop residues and animal manure, conferring them an added value. Nevertheless, further studies should be addressed in order to evaluate these results in field conditions.
